# A National Pension Fund for Hospital Officials and Nurses

**Published:** 1887-05-28

**Authors:** 


### A National Pension Fund for Hospital Officials and Nurses.

The Views of Officials, Sisters, Nurses, and others.

### By the Lady Superintendent of a Provincial Fever Hospital.

A remark made in a late issue of The Hospital *re* The National Pension Fund has somewhat reproached me, for I am one of those of whom you write—"They have almost entirely held aloof from the movement." Two years ago I sent my name to Mr. Burdett, and since then I have been waiting, and have been reading each week lately with increasing interest the various notices that have appeared in The Hospital on this subject. I can hardly express to you how intensely interested I am in all nursing and hospital work. Were I gifted with Miss Manson's power of apt expression and conciseness, I should like to write for The Hospital on many subjects. As regards the National Pension Fund, I am doing all I can with my staff (which consists of thirty nurses), and hope to send in shortly a full list of their names. I have also requested all probationers who attend my classes to write out for me their opinion on the matter. If you have any printed forms which will explain the matter more fully and clearly than I may be able to, I shall be glad to receive them, and will give them full prominence. For myself, I am entirely willing to enter the fund, for I feel that it, if established on real practical grounds, will supply a want that has hitherto been much felt by all thinking persons, and that it will moreover strengthen the links of the chain which should bind us as a nursing world together. More and more one's experience teaches that unless there is unity of aim there can be no strength in organising. To give you a short history of my nursing life, I would explain that I am a quite young superintendent, have held my present appointment two years and a half. Since the Egyptian war in 1882 I have devoted all my time to fever work; previous to that date I was taking training in all other branches of hospital work. I left the hospital I was trained at (Brownlow Hill Workhouse Infirmary) to volunteer for the Egyptian service, and undertook a six months' engagement under Government. Altogether I have been nursing nine years and a half without a break, and now begin to feel that it is somewhat trying to my health.

That I much regret not being near enough to attend the meetings of the Association, I need hardly say. I feel only half a member; but I greatly rejoice in The Hospital, and have with much interest followed the present subject under discussion in the "Public and Private Nursing" column. I entirely agree with Miss Manson, only I would go further, or perhaps I may say on another branch of the same line, were I to express my views, as they would point to the need there is that fever hospitals should train nurses for private work. There can be no doubt that this is a necessity that I believe will presently receive more notice than it does at present. That the public suffer under the existing system of sending out nurses inefficiently trained in fevers, there can be little question, in my opinion; but it is a subject that I must not begin to write about, or I may be tempted to try even an Editor's patience. Only, if you think the discussion of it might sometime suitably fall into The Hospital pages, I shall be very glad to give you my views, if I can manage the grammar! Forgive me if I have taken up too much of your time.

---

### From the Lady Superintendent, Royal Berks Hospital, Reading.

About ten of our nurses are waiting for the terms of the Pension Fund to be definitely before them, and hope then to be able to join. They all seem anxious that their own payments, or at least a portion of them, should be returned if from any change of circumstances a pension should not be required. Many of our nurses marry, and most of them have a very natural wish to have a home of their own; and a small sum in the savings-bank is more likely to assist them to the furtherance of their wishes than a better provision for a more remote time; but I think they would willingly pay £5 a year towards a pension, if there was a prospect of being able to get some of it returned. I shall be pleased to collect and forward their payments, and to advise probationers to join, if a good plan is set before them. I think it is a most desirable thing that something of the sort should be established, though in sixteen years only a very small number of our nurses have become eligible for such a provision, and I have been able to obtain it for them from our own funds.

---

### From a Trained Nurse.

I very gladly enclose my card, and should be very pleased indeed to help in any way to further the National Pension Fund for Nurses. I have read with interest the last few weeks all the correspondence on this subject, and I think all nurses must be grateful to you for the kind interest you are taking in our welfare. I am a private nurse, and have been nursing on my own account the last three years. Although I am successful and careful, I know I could never save enough to live on when past nursing. And I think it would be one of the greatest advantages in modern nursing to know we could be provided for, or at least helped, when no longer able to be of service either in public hospitals or private work. I agree with the matron of the Beckett Hospital—*i.e.*, a weekly sum to be paid to nurses when ill; a fixed sum to nearest relative in case of death; and nurses who are willing to pay sufficient now in helping to establish the fund, and who are willing to go on paying a certain fixed amount to have so much allowed them when no longer fit for our work. Allow me to say I do not consider any nurse fit for such anxious and responsible work after fifty years of age. I am afraid you will find the forming of the National Pension Fund very uphill work. I wish you every success, and shall always be much interested in any scheme connected with the Fund.

